# Microbiome in Bladder Cancer: A Systematic Review

**DOI:** 10.3390/diagnostics13010084

**Published:** 2022-12-28

**Authors:** Yong-Nam Gwon, Jae-Joon Park, Ki-Soo Lee, Kong-Hee Lee, Tae-Hyo Kim, Jae-Heon Kim

**Affiliations:** 1 Department of Urology, Soonchunhyang University Seoul Hospital, Seoul 04401, Republic of Korea; 2 Department of Urology, School of Medicine, Donga University, Busan 49201, Republic of Korea; 3 Zenit Urology Clinic, 595, Woni-daero, Seongsan-gu, Changwon 51436, Republic of Korea

**Keywords:** microbiota, urinary bladder neoplasms, systematic review

## Abstract

Although many studies on bladder cancer and the microbiome have been conducted so far, useful strains at the species level have not yet been identified. In addition, in the case of urine studies, methodological heterogeneity is too great, and in tissue studies, the species level through shotgun analysis has not been revealed, and studies using stool samples have provided only limited information. In this review, we will review all the microbiome studies related to bladder cancer so far through a systematic review.

## 1. Introduction

Human microbiota plays a crucial role in the health and development of various diseases. The significance of human microbiota is demonstrated through the attention it receives with technological advances [1,2,3]. Microbiota denotes bacteria, viruses, fungi, and archaea [1,2,4,5]. Microbiome, in contrast, is defined as the totality of genes and genomes of microbiota [1,2,4]. Microbiota hosts and microbiome maintain symbiotic equilibrium. Maintaining this equilibrium is essential for understanding the role of the microbiome in the pathogenesis of various diseases [1,4]. Developments of modern culture and sequencing technology have provided vital information on the composition and taxonomy of the human microbiome, and have enhanced the understanding of the composition and function of microbiomes in the normal state [4,6]. Consequently, studies on microbiome composition changes that result in diseases, such as cancer, are being actively conducted. Microbial dysbiosis is presumed to be caused by several stress factors, including environmental change, dietary changes, age, and smoking, leading to many diseases [1,4,7]. In particular, the mechanism by which the microbiome influences cancer pathogenesis is thought to involve the formation of metabolites such as reactive oxygen and nitrogen species. These metabolites can induce tumorigenesis by causing DNA damage via chronic inflammatory mediators [1].

Bladder cancer is a major public health problem with high socio-economic costs associated with its treatment, in addition to its post-treatment management and monitoring [8,9]. Nevertheless, prevention strategies and patient management are difficult since the etiology of bladder cancer is poorly understood [8,9]. As traditional risk factors, such as smoking, chemical carcinogens, sex, and hormones, are unable to explain the development of bladder cancer thoroughly, further research on its etiology is warranted [9,10,11,12].

The role of the human microbiome in the pathogenesis of bladder cancer has been actively researched recently. Likewise, this study performed a meta-analysis and systematic reviews of previous studies to identify the relationship between bladder cancer and the microbiome.

## 2. Materials and Methods

### 2.1. Literature Search

A comprehensive literature search was performed using PubMed/Medline, Embase, and Cochrane Library up to March 2022. The search included the following terms: relevant variants of “urinary bladder neoplasms”, “bladder cancer”, and “microbiota”. Two authors (YNG and JJP) used the inclusion and exclusion criteria to independently review the titles and abstracts of identified studies. Disagreements were resolved through discussion with a third reviewer (JHK).

### 2.2. Inclusion and Exclusion Criteria

The study’s eligibility was evaluated according to the Preferred Reporting Items for Systematic Reviews and Meta-Analyses (PRISMA) guidelines [13]. Duplicate studies were eliminated, and titles and abstracts were screened according to the eligibility criteria mentioned below. The full texts of the remaining studies were screened for inclusion using the predefined criteria. 

Studies that met the following criteria were included in this review: (1) studies including bladder cancer and control groups (case-control studies), (2) studies that provided information on the presence or abundance of microbial taxa, and (3) studies that provided information on promoting organisms or suppressing organisms in bladder cancer and/or control groups. The following studies were excluded: (1) review articles and (2) studies carried out on animals.

### 2.3. Data Extraction

Two authors (YNG and JJP) independently extracted the data using a predesigned form. Conflicts between the two authors regarding the extracted data were resolved through consensus. Extracted data from translational research included the author, study year, journal, study nationality, study population, type of specimen, cancer stage, microbiota analysis technique, type of target organisms, type of host, host sequence, promoting and suppressing organisms, alpha or beta diversity, linear discriminant analysis (LDA), and pathway analysis

## 3. Results and Discussion

### 3.1. General Characteristics of Bladder Cancer

Bladder cancer is a common cancer, with over 500,000 new cases and approximately 150,000 deaths worldwide each year [8,9,14,15]. Bladder cancer is the 10th most diagnosed cancer globally, and it is 3.7 times more common in men than in women [8,9,16]. This male-dominant tendency in the incidence of bladder cancer is presumed to be due to exposure to smoking and chemical carcinogens, which were previously identified risk factors for bladder cancer, were higher in males, and endocrine differences between the sexes [9,17,18]. Despite the current marked increase in smoking among women, there is a consistently low incidence of bladder cancer in women. Therefore, it can be inferred that there are factors affecting the development of bladder cancer in addition to previously established risk factors [9,19]. Since catabolites are excreted through the urinary tract, the diet may play a pivotal role in bladder carcinogenesis. Recently, Aveta et al., 2022 [20] reviewed the influence of meat consumption on bladder cancer incidence. The most potent mechanism involves the formation of chemical carcinogens during meat cooking and processing. Red meat and processed meat contain pro-carcinogenic compounds that are transformed into carcinogens, such as heterocyclic amines and polycyclic aromatic hydrocarbons, during high-temperature or open-flame cooking [21]. This review identified meat as a possible risk factor for bladder cancer [20]. Further studies are needed to find more risk factors associated with dietary for bladder cancer.

Bladder cancer is classified as non-muscle invasive bladder cancer (NMIBC) and muscle invasive bladder cancer (MIBC) [14]. A high proportion of NMIBCs are classified as Ta or T1, for which the standard treatment is transurethral resection of bladder tumor (TURBT). For intermediate-risk or high-risk disease, intravesical immunotherapy (Bacillus Calmette-Guerin (BCG)) or intravesical chemotherapy (mitomycin C) is immediately performed after TURBT [14,22,23,24]. NMIBC is an expensive cancer that requires periodic cystoscopy and sometimes additional tests such as urine cytology due to its high recurrence rate and the possibility of progression to MIBC [14,22,23,24]. Treatment of MIBC includes surgical therapy such as TURBT or radical cystectomy, chemotherapy, and radiotherapy, and the management cost is high and affects the quality of life of patients [14]. For this reason, there is a need for early diagnosis of bladder cancer and the development of new treatments [14]. Therefore, research on the microbiome is being conducted to elucidate factors influencing the occurrence and progression of bladder cancer.

### 3.2. Relationship between Bladder Cancer and Urinary Tract Infection

Several studies have been conducted on the effects of uropathogens on microbiota in bladder cancer. Several studies on the effect of urinary tract infection on the development of bladder cancer have shown conflicting results [9]. Numerous epidemiological studies have estimated that urinary tract infection was a major factor in the carcinogenesis of bladder cancer, but several other studies reported a lower incidence (26%) of bladder cancer in the group with urinary tract infection [25,26,27,28]. 

### 3.3. Urinary Microbiome in Patients with Urological Disease

Healthy urine has been considered sterile since the 19th century, when microbiologists discovered that sealed urine did not become turbid [14,29,30,31]. However, with the development of modern culture and sequencing technology, the detection of microbes in the urinary system has become possible, and the “sterile concept” mentioned above is being reestablished [9,14,31,32,33,34]. Due to the development of these technologies, studies are being conducted on the relationship between the occurrence of various urological diseases and the urinary microbiome. Miyake et al., 2022 [35] recently reviewed about the association of microbiome and prostate disease. In patients with prostate cancer, the positive rate of Mycoplasma genitalium was revealed higher than the rate of BPH patients [35]. One of the latest studies about biomarker is urinary levels of 8-hydroxy-2-deoxyguanosine (8-OHdG) and 8-iso-prostaglandin F2α (8-iso-PGF2α) [36]. This study showed patients with prostate cancer were significantly higher level of 8-OHdG and 8-iso-PGF2α than control group and post-prostatectomy group, whereas the control group and post-prostatectomy group showed no significant difference [36]. These findings support the hypothesis that the microbiome plays a key role in developing urological diseases, particularly bladder and prostate cancer. Current studies are being conducted to understand how the urinary microbiome affects the onset of bladder cancer and how it affects therapeutic efficacy (anti-cancer immune response) [14,37,38,39].

### 3.4. Analysis of Included Studies

**Figure 1:**
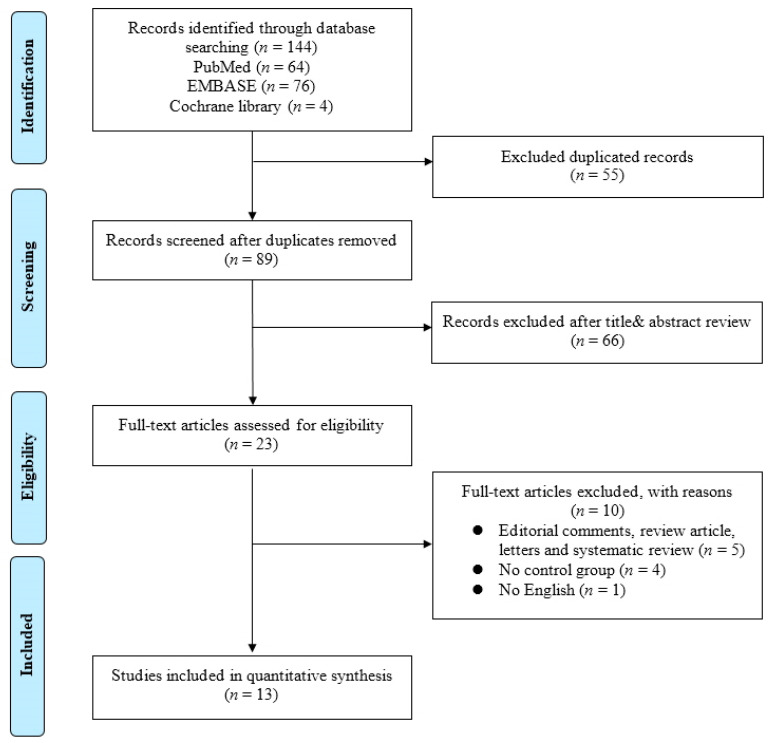
Flowchart of Preferred Reporting Items for Systematic Reviews and Meta-analysis (PRISMA).

In this study, a meta-analysis and a systematic review were performed on existing studies based on the association between bladder cancer and the microbiome. The systematic review process is summarized in a PRISMA flow diagram shown in Figure 1.

An initial literature search identified 144 studies on 7 February 2022. After duplicates were removed, the titles and abstracts of 89 articles were reviewed according to the inclusion and exclusion criteria. Then, a full-text review of the remaining 23 articles was conducted. Finally, 13 translational studies were included in the present study. Detailed information on the included studies is shown in Table 1.

A systematic review was conducted on all studies focused on the association between the microbiome and bladder cancer, and 13 were included. The types of analyzed samples, research methods, and evaluation methods showed significant differences among the included studies. Most of the included studies used urine samples, but four used tissue samples and fecal samples were used in one study. Among the four studies with tissue samples, two were performed with only tissue samples, while the other two were performed with both urine and tissue samples. Li et al., 2021 [40] focused on epithelial–mesenchymal transition (EMT); therefore, there were no results of normal tissue, only from tumors. Liu et al., 2019 [41] compared cancer tissues and normal tissues paired with cancer samples from the same patients. Mansour et al., 2020 [42] compared the microbiota composition in urine and tissue, but all tissue samples were obtained from bladder cancer. Finally, Pederzoli et al., 2020 [43] used paired triplets of urine, neoplastic, and non-neoplastic tissue specimens. However, urine specimens may be more inaccurate than tissue specimens. Additionally, only two studies used normal tissue samples paired with cancer tissue samples from the same patient. Therefore, further studies using paired tissue samples are required to obtain more reliable results. 

The 16S RNA target gene approach was used in all included studies. Whole-genome shot-gun sequencing has not been performed in bladder cancer-related analyses to date.

Diversity evaluation was divided into alpha and beta diversities and was performed in various ways. Alpha diversity was performed in 10 studies, but the presence or absence of significant differences varied inconsistently between the studies. Beta diversity analysis was performed in nine studies, and significant differences were detected between the bladder cancer and control groups in three studies. LDA analysis was performed in four studies, and pathway analyses were performed in two studies.

In a study conducted by Pederzoli et al., 2020 [43], scores of taxonomic biomarkers down to the genus level were identified by LDA using Linear discriminant analysis effect size (LEfSe) in the urine of healthy men compared to men with bladder cancer and healthy women compared to women with bladder cancer. The genera that scored ≥3 in male urine were *Tissierellaceae*, *Alphaproteobacteria*, *Rhizobiales*, *Sphingomonadales*, *Pasteurellales*, *Sphingomonadaceae*, *Pasteurellaceae*, *Streptococcaceae*, *OD1*, *and ZB2*. The genera that scored ≥3 in female urine were *Betaproteobacteria*, *Burkholderiales*, *Pseudomonadales*, *Comamonadaceae*, *Moraxellaceae*, *Coriobacteriaceae*, *Coriobacteriales*, *Coriobacteriia*, *Tepidimonas*, *Psychrobacter*, *Pseudomonadaceae*, *Xanthomonadales*, *Acinetobacter*, *Clostridiaceae*, *Procabacteriales*, *and Clostridium*. In a study by Chipollini et al., 2020 [44], LEfSe was used to identify microbial components with more abundant sequences. Significantly enriched taxa were found in the control (*Bacteroides*, *Lachnoclostridium*, *and Burkholderiaceae*) and cancer samples (*Bacteroides and Faecalbacterium*). In a study by Mai et al., 2019 [45], LEfSe was used to analyze bladder cancer and healthy control samples. *Acinetobacter(g)*, *Rhizobiales(o)*, *Enterobacter(g)*, *and Lactococcus(g)* had significant abundances in cancer samples compared to the control group with an ≥3 LDA score. In contrast, *Veillonella(g)*, *Peptosterptococcaceae(f)*, *Halomonas(g)*, *Chloroflexi(p)*, *and Dokdonella(g)* were more abundant in the control samples with ≤-3 LDA score. According to Wu et al., 2018 [46], *Acinetobacter*, *Anaerococcus*, *and Sphingobacteriaceae* were enriched taxa in the cancer group with an LDA score of ≥3; otherwise, *Bacteroidetes*, *Serratia*, *Proteus*, *Acetobacteraceae*, *Rhodospirillales*, *Roseomonas*, *and Burkholderiaceae* were abundant in the non-cancer group with ≤-3 LDA score.

**Figure 2:**
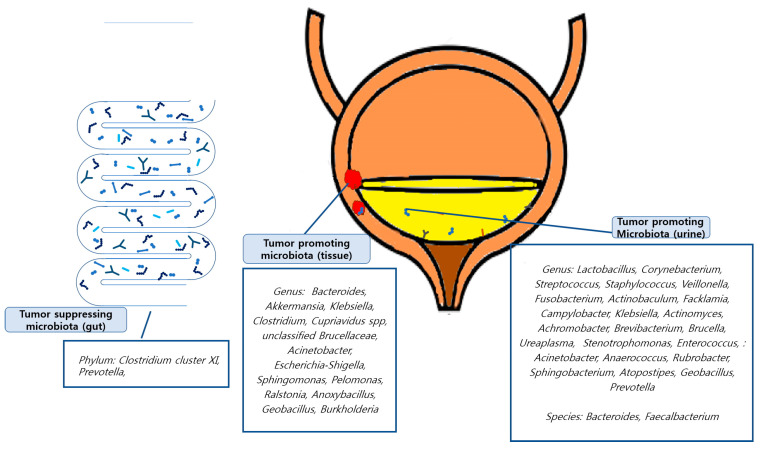
Graphic abstracts about tumor promoting/suppressing microbiota.

Tumor-promoting or tumor-suppressing organisms down to the genus level identified in the included studies were as follows: In a study conducted by Mansour et al., 2020 [42], *Bacteroides*, *Akkermansia*, *Klebsiella*, and *Clostridium* were found to be tumor-promoting organisms in tissue samples, and *Lactobacillus*, *Corynebacterium*, *Streptococcus*, and *Staphylococcus* were found in urine samples. Li et al., 2021 [40] revealed that *E. coli*, butyrate-producing bacterium SM4/1, and a species of *Oscillatoria* were associated with the expression of classical EMT-associated genes. Liu et al., 2019 [41] found that *Cupriavidus* spp., unclassified *Brucellaceae*, *Acinetobacter*, *Escherichia-Shigella*, *Sphingomonas*, *Pelomonas*, *Ralstonia*, *Anoxybacillus*, and *Geobacillus* were abundant in cancer samples and found that the tumor-suppressing organisms *Lactobacillus*, *Prevotella-9*, and *Ruminococcaceae*. Oresta et al., 2021 [47] reported that *Veillonella* was increased in pTa/T1 high-grade tumors, carcinoma in situ, and T2 tumors and *Corynebacterium* was increased in high-grade NMIBC. A significant decrease was observed in *Ruminococcus 1* and an unclassified genus of *Enterobacteriaceae*. In a study by Bučević et al., 2018 [48], *Fusobacterium*, *Actinobaculum*, *Facklamia*, and *Campylobacter* genera and two OTUs belonging to the Ruminococcaceae family were identified as tumor-promoting organisms. *Veillonella*, *Streptococcus*, and *Corynebacterium* were identified as suppressing organisms. Pederzoli et al., 2020 [43] reported that *Klebsiella* was more abundant in female urine and that *Burkholderia* was more abundant in neoplastic tissue. In a study by Hussein et al., 2021 [49], *Actinomyces*, *Achromobacter*, *Brevibacterium*, and *Brucella* were enriched in the cancer group, while *Salinococcus*, *Jeotgalicoccus*, *Escherichia-Shigella*, *Faecalibacterium*, *Thermus*, and *Lactobacillus* were abundant in the control group. Comparing NMIBC to MIBC, *Cupriavidus* was more abundant in the NMIBC group than in the MIBC group; otherwise, *Haemophilus* and *Veillonella* were more abundant in the MIBC group. Bi et al., 2019 [50] reported *Actinomyces* as a tumor-promoting organism, whereas *Streptococcus*, *Bifidobacterium*, *Lactobacillus*, and *Veillonella* as tumor-suppressing organisms. In a study by Chipollini et al., 2020 [44], *Bacteroides* and *Faecalbacterium* were promoted in the invasive cancer group, while *Bacteroides*, *Lachnoclostridium*, and *Burkholderiaceae* were promoted in the healthy control group. The superficial cancer samples did not yield any biomarker taxa. Mai et al., 2019 [45] reported that *Enterobacteriaceae*, *Streptococcus*, *Lactobacillus*, *Ureaplasma*, *Corynebacterium*, *Stenotrophomonas*, *Enterococcus*, and *Staphylococcus* were relatively more abundant in urine samples from the cancer group than in healthy control samples from other laboratories. Wu et al., 2018 [46] showed significant increases in the genera (*Acinetobacter*, *Anaerococcus*, *Rubrobacter*, *Sphingobacterium*, *Atopostipes*, and *Geobacillus*) in the cancer group and (*Serratia*, *Proteus*, *Roseomonas*, *Ruminiclostridium-6*, and *Eubacterium–xylanophilum*) in the non-cancer group. Zeng et al., 2020 [51] reported that *Staphylococcus*, *Streptococcus*, *Prevotella*, and *Corynebacterium-1* were increased in the recurrence group of NMIBC patients. Few studies have identified the microbiomes at the species level. These findings are summarized in Figure 2.

Bladder cancer, as mentioned above, is one of the most common cancers, and its treatment and management are associated with a high socio-economic burden for [8,14]. The low sensitivity of biomarkers currently used for bladder cancer screening contributes to the high socio-economic costs [52]. Thus, this study attempted to determine the relevance of the microbiome to bladder cancer and whether it could be applied to bladder cancer treatment and screening by integrating the results of several studies. However, studies to date have had limitations in that they had large differences in the methodology used and were inconsistent in interpreting the results. Since whole-genome shot-gun sequencing studies using tissue samples have yet to be performed during bladder cancer research, such studies are considered necessary for the future analysis of the etiologic factors for bladder cancer. Recently, inconsistencies in the taxonomic analysis have come to light; as such, these studies must be repeated to obtain more reliable results [53]. As such, existing data is still insufficient to clinically determine the microbiome’s relevance to bladder cancer. Therefore, additional studies using various methodologies are necessary.

## Figures and Tables

**Table 1:** Characteristics of studies included in the systematic review.

Author	Journal	Country	Population	Stage	Specimen	Method	Target	Host	Host Sequence	Promoting Organism	Tumor Suppressing Organism	Alpha Diversity	Beta Diversity	LDA	Pathway Analysis
Mansour 2020	Scientific Reports	Hungary	Total 10 Male 5 Female 5	NMIBC 6 MIBC 4	Tissue Urine	16S rRNA	Bacteria	Human	SSU Ref NR 99 database Kraken2 tool	Phylum (tissue): Firmicutes, Actinobacteria, Proteobacteria, Bacteroidetes, Cyanobacteria	NA	Yes	Yes	No	No
										Genera (tissue): *Bacteroides*, *Akkermansia*, *Klebsiella*, *Clostridium*					
										Phylum (urine): Firmicutes, Proteobacteria, Actinobacteria, Cyanobacteria, Bacteroidetes					
										*Genera (urine)*: *Lactobacillus*, *Corynebacterium*, *Streptococcus*, *Staphylococcus*					
Li 2021	Cancers	United States	Total 405	MIBC	Tissue	16S rRNA	Bacteria	Human	TCGA legacy archive NCBI nucleotide database Broad GDAC Firehose	*E. coli*, butyrate-producing bacterium SM4/1, *Oscillatoria*	NA	No	No	No	No
Liu 2019	Cancer Medicine	China	Total 22 (male)	NMIBC 5 MIBC 17	Tissue	16S rRNA	Bacteria	Human	Greengenes database	Phylum: *Proteobacteria* spp., *Actinobacteria* spp.	Phylum: Firmicutes, Bacteroidetes	Yes	Yes	No	Yes
										Genus: *Cupriavidus* spp., *unclassified Brucellaceae*, *Acinetobacter*, *Escherichia-Shigella*, *Sphingomonas*, *Pelomonas*, *Ralstonia*, *Anoxybacillus*, *Geobacillus*	Genus: *Lactobacillus*, *Prevotella9*, *Ruminococcaceae*				
Oresta 2021	Journal of Urology	Italy	Total 61 cancer 51 control 10	NMIBC 43 MIBC 8	Urine	16S rRNA	Bacteria	Human	SILVA database	Phylum: Firmicutes, Actinobacteria, Bacteroidetes, Proteobacteria	phylum: Firmicutes, Actinobacteria, Bacteroidetes, Proteobacteria	Yes	Yes	No	No
										Family: Corynebacteriaceae	Family: Enterobacteriaceae, Ruminococcus 1				
										Genus: *Veillonella* (pTa/T1 HG, CIS, and T2 tumors), *Corynebacterium* (High grade NMIBC)					
Bučević 2018	Scientific Reports	Croatia	Total 21 caner 12 control 11	NMIBC	Urine	16S rRNA	Bacteria	Human	Greengenes database	Family: Ruminococcaceae	Genus: *Veillonella*, *Streptococcus*, *Corynebacterium*	Yes	Yes	No	No
										*Genus: Fusobacterium*, *Actinobaculum*, *Facklamia*, *Campylobacter*					
Pederzoli 2020	European Urology Focus	Italy	Total 108 cancer male 34 cancer female 13 control male 34 control female 25	MIBC	Tissue Urine	16S rRNA	Bacteria	Human	RDP classifier	Order(urine): Opitutales in men	NA	Yes	Yes	Yes (urine)	No
										Family(urine): Opitutaceae in men					
										Class(urine): Acidobacteria-6 in men					
										Genus(urine): *Klebsiella* in female					
										Genus(tissue): *Burkholderia*					
Hussein 2021	Urologic Oncology	Egypt	Total 53 cancer 43 control 10	NMIBC MIBC	Urine	16S rRNA	Bacteria	Human	SILVA 16S rRNA reference (v132)	Phylum: Actinobacteria, Proteobacteria	Phylum: Firmicutes, Deinococcus-Thermus	Yes	Yes	No	No
										Genus: *Actinomyces*, *Achromobacter*, *Brevibacterium*, *Brucella*	Genus: *Salinococcus*, *Jeotgalicoccus*, *Escherichia-Shigella*, *Faecalibacterium*, *Thermus*, *Lactobacillus*				
										Phylum (MIBC): Firmicutes, Proteobacteria					
										Phylum (NMIBC): Proteobacteria					
										Genus (MIBC): *Haemophilus*, *Veillonella*					
										Genus (NMIBC): *Cupriavidus*					
Bi 2019	Journal of Medical Microbiology	China	Total 55 cancer 29 control 26	NMIBC 20 MIBC 9	Urine	16S rRNA	Bacteria	Human	Greengenes database	Genus: *Actinomyces*	Genus: *Streptococcus*, *Bifidobac- terium*, *Lactobacillus*, *and Veillonella*	Yes	No	No	No
Chipollini 2020	Urologic Oncology	United States	Total 48 cancer 38 control 10	NMIBC 22 MIBC 16	Urine	16S rRNA	Bacteria	Human	Silva version 132 classifier	Species (invasive cancer): *Bacteroides*, *Faecalbacterium*	Species: *Bacteroides*, *Lachnoclostridium*, *Burkholderiaceae*	Yes	Yes	Yes	No
He 2020	Asia Pacific Journal of Clinical Nutrition	China	Total 74 Cancer 40 Control 34	NMIBC 16 MIBC 10	Feces	16S rRNA	Bacteria	Human	UniFrac principal coordinate analysis (PCoA)	NA	Phylum: Clostridium cluster XI, Prevotella	No	No	No	No
Mai 2019	BioMed Research International	China	Total 24 Male 18 Female 6	NA	Urine	16S rRNA	Bacteria	Human	Greengenes database	Phylum: Proteobacteria, Firmicutes, Actinobacteria, Bacteroidetes	NA	No	No	Yes	No
										Class: Gamma-Proteobacteria, Bacilli, Actinobacteria, Mollicutes, Bacteroidia, Betaproteobacteria, Clostridia					
										Order: Enterobacteriales, Lactobacillales, Mycoplasmatales, Actinomycetales, Xanthomonadales, Clostridiales, Bacillales, Bacteroidales					
										Family: Enterobacteriaceae, Lactobacillaceae, Streptococcaceae, Mycoplasmataceae, Xanthomonadaceae, Corynebacteriaceae					
										Genus: *Enterobacteriaceae g*, *Streptococcus*, *Lactobacillus*, *Ureaplasma*, *Corynebacterium*, *Stenotrophomonas*, *Enterococcus*, *Staphylococcus*					
Wu 2018	Frontiers in Cellular and Infection Microbiology	China	Total 49 Cancer 31 Control 18	NMIBC 26 MIBC 5	Urine	16S rRNA	Bacteria	Human	SILVA database Greengenes database	Genus: *Acinetobacter*, *Anaerococcus*, *Rubrobacter*, *Sphingobacterium*, *Atopostipes*, *Geobacillus*	Genus: *Serratia*, *Proteus*, *Roseomonas*, *Ruminiclostridium-6*, *Eubacterium–xylanophilum*	Yes	Yes	Yes	Yes
Zeng 2020	Frontiers in Cellular and Infection Microbiology	China	Total 81 Cancer 62 Control 19	Initial NMIBC 51 MIBC 11 Follow-up NMIBC 40 -RE 5 -NR 35	Urine	16S rRNA	Bacteria	Human	SILVA database	Most abundant class in RE group: Bacilli, Gammaproteobacteria, Actinobacteria, Bacteroidia, Clostridia	NA	Yes	Yes	No	No
										Predominant order in RE group: Bacillales, Lactobacillales, Corynebacteriales, Bacteroidales, Pseudomonadales, Enterobacteriales					
										Predominant family in RE group: Staphylococcaceae, Streptococcaceae, Corynebacteriaceae, Prevotellaceae					
										Predominant genus in RE group: *Staphylococcus*, *Streptococcus*, *Prevotella*, *Corynebacterium_1*					

LDA: linear discriminant analysis, NMIBC: non-muscle-invasive bladder cancer, MIBC: muscle-invasive bladder cancer, RE: recurrence.

## Data Availability

Not applicable.
